# Reasons to be fussy about cultural evolution

**DOI:** 10.1007/s10539-016-9516-4

**Published:** 2016-02-29

**Authors:** Olivier Morin

**Affiliations:** Max Planck Institute for the Science of Human History, Minds and Traditions Research Group, Jena, Germany; Social Mind Center, Central European University, Budapest, Hungary

**Keywords:** Cultural attraction, Dual inheritance theory, Cultural selection, Imitation, Cognitive anthropology

## Abstract

This discussion paper responds to two recent articles in *Biology and Philosophy* that raise similar objections to cultural attraction theory, a research trend in cultural evolution putting special emphasis on the fact that human minds create and transform their culture. Both papers are sympathetic to this idea, yet both also regret a lack of consilience with Boyd, Richerson and Henrich’s models of cultural evolution. I explain why cultural attraction theorists propose a different view on three points of concern for our critics. I start by detailing the claim that cultural transmission relies not chiefly on imitation or teaching, but on cognitive mechanisms like argumentation, ostensive communication, or selective trust, whose evolved or habitual function may not be the faithful reproduction of ideas or behaviours. Second, I explain why the distinction between context biases and content biases might not always be the best way to capture the interactions between culture and cognition. Lastly, I show that cultural attraction models cannot be reduced to a model of guided variation, which posits a clear separation between individual and social learning processes. With cultural attraction, the same cognitive mechanisms underlie both innovation and the preservation of traditions.

Replying to a book review is rather unusual; even more so when it is as generous and perceptive as Andrew Buskell’s critique of *How Traditions Live and Die* (Buskell 2015; Morin 2016). Buskell’s essay presents important challenges for my book’s claims. Some of his concerns echo those that have been voiced by Alberto Acerbi and Alex Mesoudi, also in this journal (Acerbi and Mesoudi 2015). Both articles present, with great sympathy and care, a research trend known today as “cultural attraction theory” (Sperber 1996; Claidière et al. 2014). Cultural attraction is a way of seeing cultural change that tries to take individual cognition as seriously as possible. It shares many features with other theories of cultural evolution (those of Robert Boyd, Peter Richerson, Joseph Henrich, or Alex Mesoudi); yet its proponents (myself included) have also gone against the grain of much cultural evolution research, by criticising the use of selectionist models to study cultural change; by having ostensive communication, argumentation, or selective trust, replace teaching and imitation as the chief engines of cultural transmission; or by challenging models of cultural group selection.

Buskell, Acerbi and Mesoudi all concur that cultural attraction theorists make some valid points, but they also agree that is not as different or special as it takes itself to be. Had we paid better attention to other approaches, we would find that many of our concerns have already been successfully addressed in the cultural evolution mainstream. In particular, both Buskell’s paper (2015) and Acerbi and Mesoudi’s (2015: 485) invite us to seek agreement with Boyd and Richerson’s theoretical framework, whenever possible. I take them as sensible invitations to gloss over superficial differences, in the interest of a young discipline in need of cohesion.

I will consider three points of concern for Buskell or Acerbi and Mesoudi. On these three points, I believe that cultural attraction theorists have made an original contribution that usefully complements other approaches. Our critics doubt this. These three issues are the role of imitation in cultural transmission, the impact of cognitive constraints on culture (“content biases” in Boyd and Richerson’s framework), and the importance of transformation, as opposed to selection, in cultural change (which our critics argue can be reduced to “guided variation”).

## Does it matter whether cultural transmission is imitative or not?

*How Traditions Live and Die* is an attempt to show how the stability of human traditions can be explained without resorting to what the book calls “the Imitation Hypothesis.” According to this view, the long-term survival of traditions is chiefly due to their faithful copying and retention by human minds. I argue instead that general-purpose capacities for faithful imitation or retention are neither necessary nor sufficient for traditions to emerge. The Imitation Hypothesis is an abstraction: I do not tie it to any one author in particular, although it is closely inspired by the work of the maverick genius Gabriel Tarde (Tarde 1903), the book’s main target and inspiration. Michael Tomasello defended something very close to it in the 1990s (Tomasello 1999); whether he still does now is unclear. His work sparked a long and successful research program in comparative and developmental psychology, based (at least at first) on the view that capacities for faithful (or “true”) imitation, or faithful teaching, were something no cultural species could do without. More recently, work on “overimitation” has popularised the view that the undiscerning reproduction of pointless gestures had a deep connection with the rise of human cultures (Whiten et al. 2009). One reason to think so is that important technological innovations would have first appeared opaque or incomprehensible, and would not have been retained by discerning imitators (Csibra and Gergely 2011). All these research trends point at a process of exhaustive copying as the main driver of cultural continuity. It has other arguments in its favour: for instance, the continuity of biological lineages is arguably based on a high-fidelity, exhaustive and relatively blind replication mechanism. The Imitation Hypothesis is no straw man, nor is it wrong in any obvious way.

I am not the only one to doubt it, though. The book builds on the work of modellers and theorists who show that robust cultural transmission need not be based on a process that reproduces ideas or behaviours with high fidelity, for three main reasons. First, cultural diffusion can do with a lot of waste: stable traditions may still emerge if most of what we do or say is badly distorted or not copied at all (Sperber 1996). Second, repeated exposure to a cultural trait can produce robust transmission (especially when a variety of sources are involved), even when each single transmission event is error-prone (Strimling et al. 2009; Eriksson and Coultas 2012).^1^ Lastly, some cultural forms are cognitive “attractors”: they are intuitive enough to emerge spontaneously in many different cultures, and they are readily reconstructed if distorted (Sperber and Hirschfeld 2004). Together, these three mechanisms paint a picture of cultural transmission that is radically different from the one depicted by the Imitation Hypothesis, from Tarde on. The key difference is that transmission does not have to rest on mechanisms whose evolved function or habitual activity is high-fidelity copying. High-fidelity copying certainly happens, but it is neither necessary nor sufficient to the stability of cultural forms.

In the three processes just listed, the fit between human minds and human traditions plays a key role. They need to be appealing to many people, they need to elicit frequent production and repetition, and they need to be easily reconstructed. We believe that, in this respect, some cultural forms are luckier than others. Here is an analogy. Suppose a photocopier is defective: it systematically produces copies that are darker than the original. You write up a note, make a copy of it, take that copy, make a copy of it, and so on, feeding each copy back into the photocopier. After a certain number of copies, you will end up with an entirely black sheet of paper. You go on making copies of it. At this point, something interesting happens: the photocopier stops being defective. The copies of the black sheet that it churns out are perfectly similar to their model. Someone entering your office at this point could reasonably assume that the photocopier is perfectly functional—but it isn’t: it only works well for one very special kind of input. In the same way, the fluency of cultural transmission in our species could easily lead observers to believe that we are born imitators, and that our species’ cultural success rests on this ability; but that is not the only possible explanation. We might be good at transmitting culture because it fits some of our mind’s proclivities: because it is like the black sheet of paper.

Buskell read *How Traditions Live and Die* as an attempt to use the argument above to refute in bulk the contributions of Robert Boyd, Pete Richerson and Joseph Henrich to the field of cultural evolution. That was certainly not my intention. These authors’ contribution to the field fully deserves its prominence. My book discusses their work but I also praise it (e.g., Morin 2016: 88–91), and repeat it on many points. I agree with Buskell that many modelling results of theirs will still stand even if we discard the Imitation Hypothesis.^2^

This is not going quite far enough for Buskell. He invites me to take a purely instrumental view of the work of Boyd, Richerson, and Henrich. It consists at its core (Buskell argues) in abstract models. These models can be “unpacked”: they can be made to relate to empirical phenomena; but such unpacking is complex, fraught with difficulty, and ultimately secondary. If modellers unpack their work in a way that is debatable or contrary to facts, we should not blame them, but rather try to find better ways of unpacking. It is in the nature of multi-agent models to gloss over important psychological issues, and the resulting simplifications cannot be held against them. The way I read Buskell, when a claim is made in relation to a set of models, it can only be criticised by showing how its being false could affect the models in question. Merely saying the assumptions are wrong is a cheap shot: all models are somewhat wrong—but some are useful.

Should Buskell’s advice be followed? One of the things I most admire with Boyd, Richerson and Henrich’s work is their consistent refusal to insulate their models from reality. Cultural evolution could easily go the way of neo-classical economics: abstract constructions drifting away from testability. That this is not happening is to be put to their credit. Theirs is a beautifully integrated theory of cultural evolution, where models mesh with archaeological, ethnographic, and experimental data. One part of this theory is a complete account of the psychology of cultural transmission. This account was not put forward reluctantly, as a convenient simplification good enough to feed a model. They have defended it with enthusiasm and sought experimental and ethnographic validation for it, often with considerable success. We should take them seriously when they claim that only imitation and teaching, but not weaker forms of social learning, can sustain cultural evolution (Boyd and Richerson 1985: 34–35); that cultural evolution requires specific, naturally selected adaptations for accurate cultural learning (Boyd and Richerson 1995: 82; Henrich 2015); that people are natural, automatic, unconscious imitators of others (Henrich 2015). Such claims are worth discussing on their own, independently of their role as modelling assumptions.

## Shouldn’t we use Boyd and Richerson’s “content versus context” distinction?

Cultural attraction theorists believe that cultural evolution is driven by a myriad of widely shared tastes and preferences that bring people in many different societies to favour similar traditions. Examples range from direct-gaze portraits to counter-intuitive deities. On this point, cultural attraction gets two kinds of criticism.

Skeptics (whose views are echoed in Buskell’s review) note the rashness of making universalistic claims about anything psychological, when our typical experimental sample consists of a few Western undergraduates. As Buskell notes, no cultural form is so universally appealing as to be preferred by everyone in all circumstances (as far as we know). He rightly notes that my discussion of “extreme traditions” (2016: chapter 6) sometimes exaggerates the power of psychological attraction. Yet, at bottom, the view of cultural longevity defended in the book does not depend on such excessive assumptions (the occasional overstatement notwithstanding). All it requires is that some traditions enjoy a comparative advantage over others, for most people, in a wide variety of contexts. The evidence for this is strong enough, I think, to shift the burden of proof onto the skeptics’ shoulders. Few would deny that salty food has an edge over saltless food—not a universal edge or a systematic one, but more than enough to account for the enduring popularity of salt. Less trivially, several studies show that direct-gaze babies are attracted to direct-gaze faces (as opposed to averted-gaze ones), a preference that is maintained in adulthood. Again, this preference is neither systematic nor truly “universal”; yet the cultural importance of the direct gaze (even in places where it is tabooed) is difficult to explain away without taking this into account.

On the other side we have believers, like Acerbi and Mesoudi, who agree that some cultural forms are quite generally appealing, but who point out that cultural attraction theory is not the only framework in which to investigate species-general cognitive biases, as several remarkable studies have demonstrated (Henrich and Henrich 2010; Mesoudi et al. 2006; Eriksson and Coultas 2012; Xu et al. 2013). Acerbi and Mesoudi also fault cultural attraction theorists for not paying proper attention to the work of Boyd, Richerson and Henrich. In their framework (Richerson and Boyd 2005), two basic sources of cultural appeal are distinguished. On the one hand, we have “context biases”, of which the most important are prestige and conformity biases (the appeal of cultural features seen among the prestigious or the many). On the other hand, we have “content biases”, which may indeed include innate or universal preferences. Since no one would disagree that both “content” and “context” biases play a role in cultural change, why be fussy about it?

There is a saying that scholars would sooner exchange underwear than terminologies. A little more, I think, is at stake here. The context/content dichotomy invites us to single out imitation of the prestigious or the many as two of the most important drivers of cultural evolution. I am reluctant to do so because I believe that these ideas carry a fundamental ambiguity, not just in our field, but in social science and social psychology since the days of Tarde. My book tries to show that Tarde’s notion of “prestige” was marred by conceptual confusions that have persisted through the twentieth century and to this day, and that a focus on conformity often prevents us from exploring alternative explanations that are just as plausible (Morin 2016: chap. 3).

Boyd and Richerson’s context biases can be “unpacked” in two distinct ways. On the one hand, contextual information can be seen as a set of cues that humans use flexibly and discriminately to inform their cultural decisions. I assume that this is what most readers think of when they read the phrase “conformity bias” or “prestige bias”: they assume that people use social cues, in combination with other cues, to make cultural decisions. Occasionally these cues are so strong, and other cues so ambiguous, that the prestige or number of models carries the day, but most of the time our minds avail themselves of most useful and available cues, whatever they are. The book defends this view, arguing that “context biases” only trump psychological attraction when attraction is weak (for instance, the prestige of famous Renaissance painters did not prevent younger painters from producing more direct-gaze portraits than their elders—Morin 2013). The book also argues that contextual cues can be much less useful and less accessible in reality than in models: evaluating a hunter’s performance accurately, for instance, is no easy task (Hill and Kintigh 2009), and prestige may not be a reliable guide to competence (Reyes-Garcia et al. 2008).

A stronger interpretation would hold that contextual cues form the basis of simple decision rules, which use these cues in a mechanical fashion to form simple heuristics. This problematic interpretation of “context biases” is not, it is true, the only one, but it is the one that has been pushed most consistently by Boyd, Richerson, and Henrich, as I interpret them (Richerson and Boyd 2005; Henrich 2015). The prestige or number of models, in this view, is not simply one cue among others. It is a type of cue that triggers a special kind of decision mechanisms. These mechanisms follow social cues (like number or prestige), but they ignore other cues even when these might be more informative.

Once again, this proposal is quite defensible. Boyd, Richerson and Henrich’s strongest argument (as I read them) is that our reliance on context-biases is a “simple heuristic that make us smart” (Gigerenzer’s phrase) (Richerson and Boyd 2005: 119–120). We know from Gigerenzer’s work that such heuristics can sometimes outperform more information-hungry decision rules. Context biases might be like this: restricted (in the range of cues they process), and thus more efficient. There is a problem with this argument, however. Boyd, Richerson, and Henrich stress the fact that context biases often produce maladaptive decisions that other decision rules would avoid. Henrich, for instance, argues that suicides are readily imitated—celebrity suicides, but also anonymous ones (Henrich 2015: chap. 4). He takes this as a sign that our drive to imitate could override our most deeply rooted instincts—including the will to survive, and produce severely maladaptive outcomes. Contagious suicide is a familiar trope in the imitation literature since Tarde, and my book tries to show that the evidence for it is much weaker than it appears (pp. 110–115). True or not, it is hard to see how a heuristic that produces such behaviours could make us smarter than one that would also makes room for non-social learning and evolved preferences. More generally, Boyd and Richerson’s claim that social learning solves a trade-off between generality and accuracy, at the expense of accuracy (Richerson and Boyd 2005: 158–162, 188), is hard to reconcile with the spirit of Gigerenzer’s heuristics. The “simple heuristics that make us smart” normally sacrifice complexity with no loss of accuracy (Gigerenzer and Goldstein 1996).

This ambiguity—are context biases merely cues, or full-blown heuristics?—is arguably secondary in the mind of many readers of the literature. They simply opt for the weakest interpretation of context biases: the prestige and number of models are one type of cues among many, to be weighed against other elements like any piece of information. Still, there is yet another reason to be wary about the context/content dichotomy: it makes no room for hybrid mechanisms, where intrinsic cognitive appeal and social influence interact (Sterelny 2012: 28). Take argumentation. The fact that a scientific theory is backed by good arguments has much to do with the theory’s intrinsic merit (its coherence, its fit with known data, etc.); but a lot also rests on the skills of the people who argue for it, on their capacity to reach wide audiences, etc. Context? Content? Both—or neither. Trust is another case in point. Much social influence is driven by experts whose range of influence is circumscribed to a very narrow domain (see Morin 2016: 115–119 for a summary of the evidence). This implies that the choice of following a given maven on a given issue is itself influenced by a body of rather sophisticated (and domain-specific) individual knowledge. Ann is my go-to person for pop music, but I think she is biased in favour of hip-hop, and I do not quite understand her recent infatuation with K-pop. I tend to listen what she listens to, except for hip-hop, and the next time she recommends downloading something Korean, I will seek a second opinion (or listen by myself first). Likewise, a large part of scientific expertise, is a matter of knowing the right people to trust, and on which topics (Shapin 2015). Here again, a lot of “content”-relevant information enters into “context” biases.

## Shouldn’t cultural attraction be reduced to Boyd and Richerson’s “guided variation”?

One consequence of letting go of the Imitation Hypothesis is that we cannot account for the stability of traditions by positing one general drive to reproduce ideas or behaviours. We need to explain, on a case-by-case basis, why certain ideas or behaviours will fit our cognitive biases in such a way that stable diffusion chains will form. Cultural attraction theorists hold that there is no fully general answer to this question: each tradition is attractive for its own reasons. Some are attractive for local and contingent reasons (which means they won’t usually last); others owe their appeal to very widespread preferences and biases (many long-lasting traditions should be like this).

Cultural attraction theory is sometimes taken to claim that cultural transmission is generally less faithful, or more “noisy”, than is thought—or that people are more creative, less culturally determined than commonly assumed. That is not its main point. Attraction can make transmission very accurate indeed: a legend that fits our cognitive biases, a recipe that flatters a widespread taste, or a style of painting that draws attention, can be reproduced with high fidelity. The important point is that the very same mechanisms that produce faithful reproduction can also produce drastic changes, depending on the input they receive (Kalish et al. 2007; Scott-Phillips in press). The same memory processes that ensure the transmission of a cognitively attractive legend can badly distort another tale, if it does not fit. Besides, not all distortions are interesting to students of cultural attraction—only those that are oriented towards a particular attractor. Random distortions do not, in the long run, influence cultural change in this or another way, and fail to contribute to attraction. We are not making any claims about the actual amount of noise in cultural transmission; we are saying that transmission can happen in spite of the noise, because traditions get reconstructed, not simply replicated. Cultural attraction theory is entirely compatible with the view that high-fidelity transmission does occur, giving rise to forms of cultural evolution that resemble natural selection in some respects (in full agreement with Acerbi and Mesoudi).

This makes me resist the view that cultural attraction could be reduced to Boyd and Richerson’s “guided variation” (Boyd and Richerson 1985: 81 sq.). Acerbi and Mesoudi argue that it can (2015: 488). Guided variation, according to Boyd and Richerson, is a cultural equivalent of the “Lamarckian” inheritance of acquired characteristics: individual learning guides inventors to new cultural practices (with no help from others), then these practices spread in populations of learners (provided they enjoy a selective advantage over others).

Guided variation, thus conceived, allows Boyd and Richerson to make several important points that I fully agree with (so probably do Sperber, Claidière and Scott-Phillips). For a start, we agree that cultural evolution is not blind: it is shaped by human cognition, not (or not exclusively) by blind variation followed by selective retention (contra Campbell 1960). As a consequence, purely selectionist models fail to capture what goes on in cultural evolution. Selection-like processes do occur, but non-random variation and transformation also orient cultural change (see also El Mouden et al. 2014). What the exact balance between selectionist and non-selectionist forces consists in is a matter of degree, and has to be estimated on a case-by-case basis: the importance of selection versus guided variation is probably not one that admits of a general answer. Different perceptions may simply come from the fact that different researchers are considering different phenomena (with cultural epidemiology being more concerned with religion, knowledge, or oral traditions, rather than technology or institutions).

So far, so uncontroversial. Why not (Acerbi and Mesoudi ask) simply agree to agree, and call it a day? I see two reasons for that. I agree with Acerbi and Mesoudi that the first one (the one most commonly put forward) may not be compelling; yet a second reason justifies, in my view, that we keep being fussy about guided variation.

The most obvious difference between transformation and guided variation is that the “guided variation” model admits no middle ground between innovation and imitation. Social learners do not typically reinvent or transform what they copy (Boyd and Richerson 1985: chap. 4; Richerson and Boyd 2005: 161, 181). If transformations are allowed to take place, Henrich and Boyd have argued that selective imitation always trumps them (Henrich and Boyd 2002). That is one clear point of disagreement with Claidière et al. (2014) and Claidière and Sperber (2007).

However, Acerbi and Mesoudi have a point, I think, when they note that the distinction between, on the one hand, selection on guided variation, and transformation on the other hand, may often be empirically moot. That is because, as they note, the line between innovation and transformation is slender, and a difficult one to draw. One theorist’s transformation could be another’s innovation. Even more worryingly, the distinction could depend on the way cultural units are defined—a notorious ontological quagmire. Acerbi and Mesoudi note, quite perceptively, that my study of the rise of direct-gaze portraits (Morin 2013) cannot distinguish between selectionist dynamics and transformative ones. It could be described as painters selectively copying the most successful portraits (which happen to be direct-gazing, or so I argue); but it could as easily be counted as a case of evolution by transformation, with each individual painters adopting the style of portraiture of their elders, but increasing the importance of direct gaze in their own style. The fact that the answer may differ depending on the unit analysis we choose (portraits vs. painters) should indeed give us pause. We want to avoid making hypotheses that work for descriptions of a certain granularity, but not for others.

The real reason why we should (I think) resist the reduction of attraction to guided variation, is different. “Guided variation” is premised on the existence of two independent kinds of processes: individual learning generates variation, while social learning (i.e., imitation and teaching) ensures transmission. In the “guided variation” model, individual learning cannot avail itself of social information, and social learning is narrowly imitative: it can only adopt what it learns, not modify it. The more we avail ourselves of social learning, the less we can call on individual learning, and vice versa (Boyd and Richerson 1985: 81–82, 97–99).^3^, ^4^

To see why the distinction between guided variation and imitation does not carve nature at its joints, it might be helpful to go back to an example put forward by Acerbi and Mesoudi: the hand copying of manuscripts. The diffusion of ancient texts can be analysed, in Darwinian fashion, as a process of generally faithful replication coupled with various kinds of mutation (deletions, recombinations, etc.) (Howe et al. 2001). That the analogy can be applied should not be too surprising: after all, the very notion of a genetic code was built on an analogy with textual copying; yet this analogy misses a lot that is worth knowing about the processes of book copying in the Western Middle Ages, of which there were mainly two. Before the rise of solitary hand copying as we know it, much copying was probably done orally—with one monk reading the text aloud and the other copying it under dictation. Such “oral copies” contain specific mistakes (errors of the hear, not of the hand) that disappear with the rise of modern, “read” copies—which gave rise, in turn, to another specific type of mistakes (Saenger 2000: 48–51). This crucial difference is all but invisible to the genetic analogy, which works for both kinds of copying, oral and visual. In both cases there will be changes that can be described as deletions, recombinations, and mutations, along with faithful copying; but this in itself tells us little of what there is to learn about the underlying processes of transmission.

The mistakes that arose during book copying can perhaps be described as “guided variation,” if this means they were non-random innovations. Certain letters or words are much more likely than others to be transformed, and the result of the transformations is predictable, to a point. All this was worked out by scholars a long time ago (and without any help from genetic or Darwinian analogies—Reynolds and Wilson 1991). Yet, these scholars knew one thing that the notion of guided variation may cause us to miss: a great proportion of the mistakes we can observe originated as corrections or re-interpretations (Reynolds and Wilson 1991: 233–235). The “mistakes” were attempts to restore what was thought to be the original meaning, or the correct spelling. It is actually quite likely that, in many cases, this is exactly what the copyists ended up doing: using their epigraphic knowledge or their interpretive skills to rectify a mistake in their model, or one that they themselves made while reading (or hearing) the manuscript. In other words, the very same process could cause both variation and preservation; individual reinvention is not the opposite of transmission.

I should note^5^ that the opposite also happens: what the monks read and copied influenced their skills. For a first, without texts to read they would never have become literate. More subtly, it is likely that they became, every time they copied a given word they already knew, a bit more familiar with that word, and thus more likely to correct its variants to something they knew (judging by what we know from current research on the cognitive effects of repetition and use.). The interplay between cognitive mechanisms and the representations they process is not a one-way affair: what goes through our minds changes our minds. Cultural epidemiology, like evolutionary psychology, is not wedded to a rigid form of nativism: communication and culture do build partially novel mental faculties or inclinations (Barrett 2015; Sperber 2005). This admission does not take away the distinction between representations and cognitive processes (just because the two interact does not mean we cannot distinguish between them); nor do we have to agree with the view which holds that human capacities for communication mainly arose thanks to the influence of some particular cultures, and owe little to the shared biological legacy of our species (see a case for this view in Heyes and Frith 2014, and discussion in Morin 2016: 67–68).

## Conclusion

I hope to have shown that the occasional disagreements between cultural attraction theorists and others do not spring from ignorance or caricature. My interlocutors would probably reply that there is still much more in common between all of us, students of cultural evolution, than between all of us and (say) partisans of the “ontological turn” in anthropology. They would be right. Had I been writing for a less sophisticated audience, I would have stressed the unity of cultural evolution research; but philosophers know how important it can be to scrutinise a discipline’s assumptions. There may never be a better time to do this for our field: its working hypotheses have not crystallised into dogmas, and there is room for a healthy pluralism of views on such fundamental issues as the role of imitation in transmission, the importance of cognitive constraints on culture, or the impact of selectionist dynamics.

## Footnotes

1 These contributions (cited in my book) are due to researchers with no particular connection to Boyd and Richerson, who do not claim credit for this work, *pace* Buskell (Buskell 2015: 4th page).

2 For instance, when discussing the links between culture and altruism, I argue that some models—chiefly due to Herbert Gintis and Herbert Simon—, require that we compulsively imitate deleterious behaviours. The book makes it clear, I think, that this is not always true of most of Boyd, Richerson and Henrich’s cultural group selection models (Morin 2016: 95).

3 “The models in this book assume that culture has the properties of an inheritance system. (…) culture can have this property only if individual learning is not too important in determining behavior.” (Boyd and Richerson 1985: 98).

4 Theoretically, this model *could* allow individual learning to contribute to cultural stability (thanks to Alberto Acerbi for pointing this out to me). Guided variation may result in “convergent” cultural evolution (different individuals and cultures coming up with similar innovations), and also in the periodical re-invention of certain traditions, which might be lost but reinvented. However, this possibility has not, to my knowledge, been explored. Boyd, Richerson and Henrich have always insisted that most human traditions are beyond the power of unaided individual cognition to reinvent. You cannot, on your own, devise smart ways of surviving in the jungle, or building a kayak.

5 Thanks to Kim Sterelny for this remark.
